# Juvenile dermatomyositis: association between nail fold capillary end row loop– area under the curve– and disease damage indicators

**DOI:** 10.1186/s12969-023-00919-3

**Published:** 2023-11-13

**Authors:** Amer Khojah, Gabrielle Morgan, Marisa S. Klein-Gitelman, Lauren M. Pachman

**Affiliations:** 1 Department of Pediatrics, College of Medicine, Umm Al-Qura University, Makkah, Saudi Arabia; 2 Division of Pediatric Rheumatology, Ann & Robert H. Lurie Children’s Hospital of Chicago, 225 East Chicago Avenue, Box 50, Chicago, IL 60611 USA; 3 Feinberg School of Medicine, Northwestern University, Chicago, IL USA

**Keywords:** Juvenile Dermatomyositis, Nailfold vasculature, Disease Activity scores, Area under the curve, Lipodystrophy

## Abstract

**Background:**

Juvenile Dermatomyositis (JDM) is a rare autoimmune disease characterized by skin and muscle inflammation. The loss of nail fold capillary end row loops (ERL) is evidence of small vessel involvement in JDM. This study aimed to examine the specific association of ERL over the disease course with evidence of JDM disease damage.

**Methods:**

We analyzed data from 68 initially treatment-naïve JDM children who had been observed for at least five years with multiple ERL density assessments. The JDM disease course were categorized into monocyclic short, monocyclic long, polycyclic, and chronic. The ERL capillary count was cumulatively evaluated using the area under the curve (AUC) method.

**Results:**

The mean ERL density for the treatment-naive JDM was significantly lower than that of their healthy age-matched controls (4.8 ± 1.6 /mm vs. 7.9 ± 0.9 /mm; p < 0.0001). The ERL AUC was significantly lower in children with a chronic disease course compared to those with a monocyclic short (p = 0.001) or monocyclic long disease course (p = 0.013). JDM patients with lipodystrophy had lower ERL AUC than those without lipodystrophy (p = 0.04). There was no association between ERL AUC and calcifications or fractures.

**Conclusion:**

Persistently decreased ERL capillary density, reflected by low ERL AUC, is associated with a chronic disease course and lipodystrophy in JDM. Despite medical therapy, the mean ERL count remained below normal even after five years, particularly in polycyclic and chronic cases. It is not clear that restoring normal capillary density is currently feasible in children with JDM.

**Supplementary Information:**

The online version contains supplementary material available at 10.1186/s12969-023-00919-3.

## Introduction

Juvenile Dermatomyositis (JDM) is a systemic pediatric autoimmune disease characterized by skin and muscle inflammation [1]. Although it is the most common pediatric inflammatory myopathy, with an incidence rate of approximately 3.2 cases per million children in the United States, JDM remains a rare disease [2]. Its etiology is not entirely understood, but it is believed to involve a combination of specific genetic predisposition and environmental factors, such as viral infections and exposure to ultraviolet rays [3, 4].

One of the key features of JDM is the loss of nailfold capillary end row loops (ERL), which can be evaluated at the bedside using capillaroscopy [5–7]. The loss of capillary ERL indicates small vessel vascular injury, which is further supported by elevated levels of von Willebrand factor antigen (vWF antigen) in the treatment-naïve JDM patients [8]. The reduction in ERL is linked to lower bioavailability of oral prednisone compared to IV methylprednisolone making the oral route of medical therapy less effective [9]. Inadequate treatment of JDM can lead to a higher risk of complications such as calcinosis, deposition of insoluble calcium salts in the skin and subcutaneous tissue, and lipodystrophy [10, 11]. Therefore, defining the association between ERL density and disease progression is important for patient care.

This study aims to examine the association between ERL density over time (5 years) using the area under the curve (AUC) method to define the range of various diseases courses (monocyclic short, monocyclic long, polycyclic, and chronic) in addition to indicators of disease damage (lipodystrophy and calcification).

## Methods

### Subjects

This retrospective chart review study (IRB# 2012–14,858) was conducted at Ann & Robert H. Lurie Children’s Hospital of Chicago. We included all subjects with the JDM diagnosis based on Bohan and Peter criteria [12, 13] who had at least five years of follow-up data and had at least four ERL assessments at a prespecified time point (0,6,12,24,36,48, and 60 months from initiation of medical therapy). Patients who received medical therapy before the initial ERL assessments, those lacking a five-year follow-up, or those with overlap syndrome were excluded from the analysis. The JDM disease activity was evaluated using standardized scoring systems—the Disease Activity Score (DAS) [14] and Childhood Myositis Assessment Scale (CMAS) [15]. The demographic data of the JDM children are presented in Table 1.

**Table 1:** Demographic and disease characteristics of the JDM cohort

	Frequency (n)	Percentage
Sample size	68	
Sex		
Female	57	16.2%
Male	11	83.8%
Race/Ethnicity		
White	51	75.0%
Hispanic	13	19.1%
African American	2	2.9%
Others	2	2.9%
Myositis specific antibodies		
P155/140	28	41.2%
MJ	2	2.9%
Mi2	5	7.4%
MDA5	2	2.9%
Others or multiple MSAs	5	7.4%
Negative	21	30.8%
Not done	5	7.4%
Treatment		
Oral steroid	68	100%
Intravenous steroid	64	94.1%
Methotrexate	63	92.6%
Intravenous immunoglobulin	8	11.8%
Hydroxychloroquine	41	60.3%
Cyclosporine	12	17.6%
Mycophenolate	26	38.2%
Disease course		
Monocyclic short	12	17.6%
Monocyclic long	29	42.6%
Polycyclic	14	20.6%
Chronic	13	19.1%

The study included 77 healthy children as a control group after providing appropriate written consent (IRB# 2001–11,715). These healthy controls did not have any autoimmune disease or active infection at time of their enrollment. In appreciation for their participation in nailfold capillaroscopy, they received a $25 gift card. Demographic details of healthy controls are available in the supplementary materials (Supplemental Table 1).

### Disease course

Children with JDM were categorized into four distinct disease courses according to their treatment response: (A) monocyclic short: if the child completed therapy within the first 36 months without a subsequent disease flare; (B) monocyclic long: if the child completed therapy after 36 months without a subsequent disease flare; (C) polycyclic: if the child had completed treatment but had a subsequent relapse of disease requiring re-initiation of medication; (D) chronic: no clinical resolution within 60 months.

### Nailfold capillary ERL studies

Standardized images of the nailfold area were obtained using a Nikon Coolpix p6000 digital camera equipped with a Dermlite2 ProHR (18x). The analysis of the nailfold images was performed by a single experienced observer (GM) using Photoshop. The number of ERL per 3 mm section on each of the eight fingers (excluding thumbs) was counted and subsequently divided by three. Each patient’s mean ERL/mm was calculated by averaging the ERL/mm of the eight fingers [5, 16].

### ERL area under the curve calculation

**Fig. 1:**
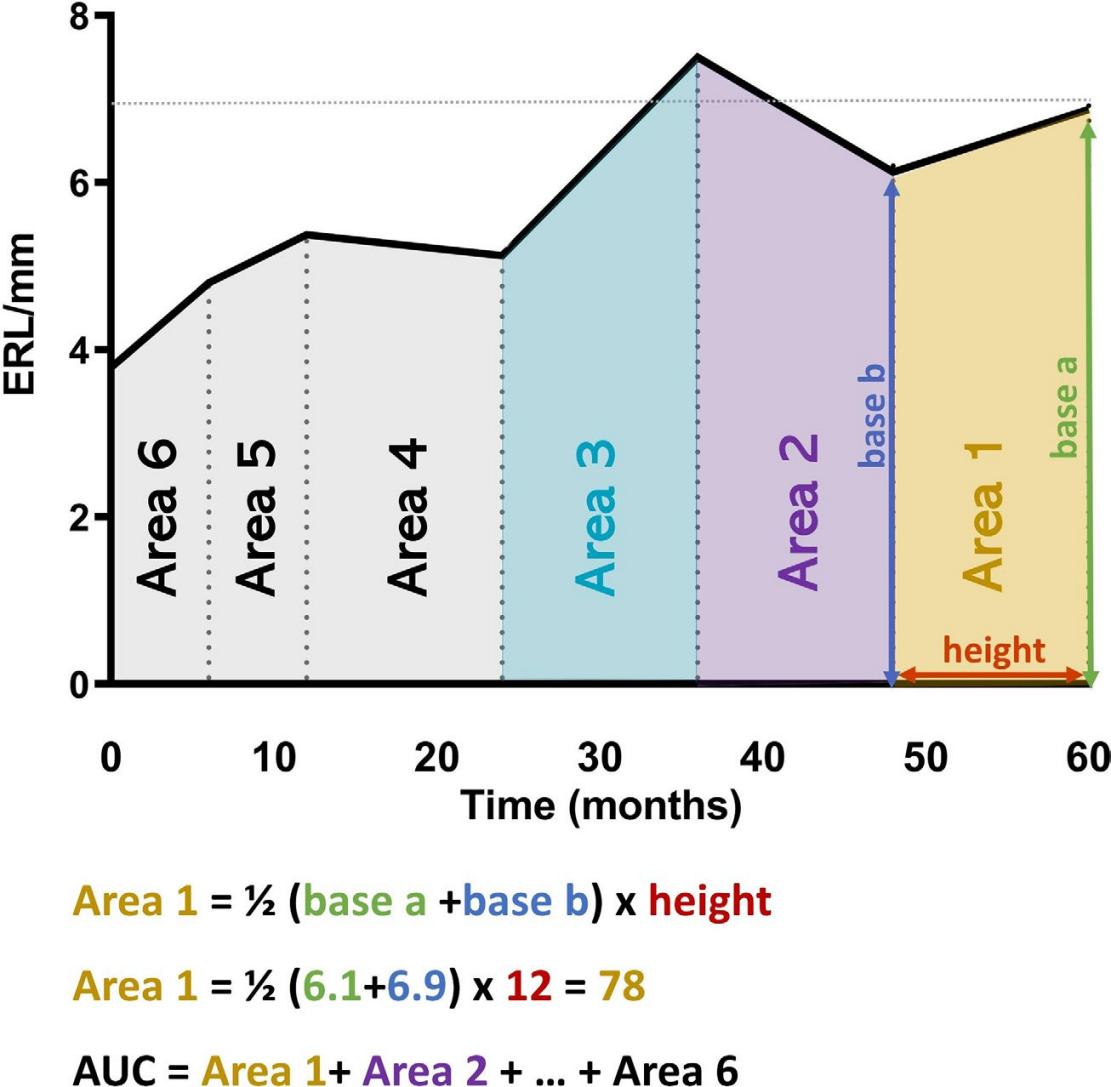
Area under the curve (AUC) calculation by GraphPad Prism. First, the curve was created by plotting the ERL data over time. Then, Prism divides AUC into multiple small trapezoid areas, which are measured individually, using the trapezoid rule [area = ½ (base a + base b) x height], and added up to get the total AUC.

GraphPad Prism was used to calculate the AUC to measure the ERL cumulatively across the study duration. First, the curve was created by plotting the ERL data over time. Then, Prism divides AUC into multiple small trapezoid areas, which are measured individually, using the trapezoid rule [area = ½ (base a + base b) x height], and added up to get the total AUC (Fig. 1).

### Statistical analysis

The Person’s correlation coefficient was utilized to assess the relationship between ERL at diagnosis and ERL AUC. The student t-test was used to compare the mean ERL AUC of subjects with and without signs of disease damage. Statistical analyses were conducted using IBM SPSS Statistics® and GraphPad Prism® version 9.4.1 was utilized to generate the figures.

## Results

The study included 68 treatment-naive JDM children, the majority of whom were female (84%). The racial and ethnic distribution was as follows: 75% Caucasian, 19% Hispanic, 3% African American and 3% Others. Their MSAs (Myositis-specific antibodies) were as follows: 41% P155/140+, 3% MJ+, 7.5% Mi-2+, 3% MDA-5+, 7.5% multiple MSAs, and 31% MSA negative (Table 1). The mean age of onset for JDM was 6 ± 3.1 years, and the mean duration of untreated disease was 9.6 ± 10.2 months (Table 2). The initial disease activity scores were 11.0 ± 3.6 for DAS total, 5.9 ± 1.5 for DAS skin, and 5.1 ± 1.5 for DAS muscle, and CMAS score was 37 ± 10.3 (Table 2). The disease course distribution was as follows: 17.6% monocyclic short, 42.6% monocyclic long, 20.6% polycyclic, and 19.1% chronic (Table 1).

**Table 2:** Baseline (before treatment) disease activity assessment of the JDM cohort

	Reference Range	Mean ± SD	Median (range)
Age (years)		6 ± 3.1	5.4 (1.9–16.4)
Duration of untreated disease (months)		9.6 ± 10.2	6.5 (1–73)
Clinical disease activity indicator			
DAS-total	0	11 ± 3.6	11.5 (3–19)
DAS-skin	0	5.9 ± 1.5	6 (2–9)
DAS-muscle	0	5.1 ± 1.5	5 (0–10)
CMAS	52	37 ± 10.3	38 (12–52)
ERL (#/mm)	> 7	5 ± 3.1	4.9 (2.3–10.3)
Laboratory disease activity indicators			
Neopterin (nmol/L)	< 10	19.4 ± 10.6	18.5 (2.4–49.3)
ESR (mm/hr)	< 20	19 ± 13.4	15 (3–65)
vWF Antigen		156 ± 75	140 (52–374)
Muscle enzymes			
CK (IU/L)	26–27	1700 ± 5291	140 (57-35471)
AST (IU/L)	17–96	107 ± 162.5	46 (22–890)
LDH (IU/L)	147–463	455 ± 392.6	342 (166–2259)
Aldolase (U/L)	3.4–8.6	21.6 ± 41.6	9.9 (2.8–237)
Flow cytometry			
Total T cells (CD3+)		64 ± 8.3	64 (41–86)
T helper cells (CD3 + CD4+)		44.4 ± 8	45 (26–64)
T cytotoxic cells (CD3 + CD8+)		18.6 ± 4.4	19 (6–31)
B cells (CD19+)		29 ± 8.3	29 (12–52)
NK cells (CD16+/CD56+)		6.3 ± 3.6	5 (1–15)

**Fig. 2:**
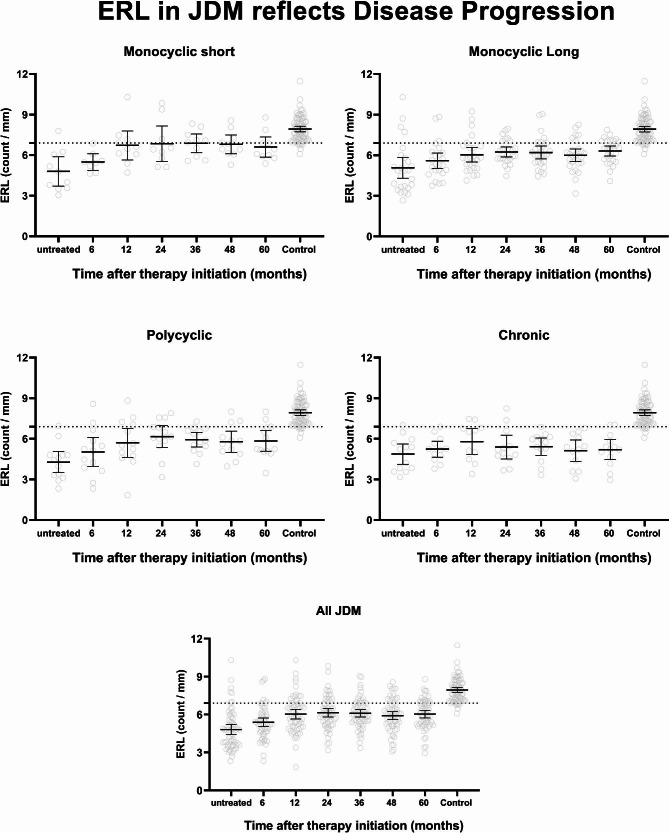
Changes in ERL capillary count over time (5 years) by disease course categories. The rate of improvement varied, depending on the different disease courses, with monocyclic short showing a faster recovery than the other groups

The mean ERL count for treatment-naive JDM was 4.8 ± 1.6/mm, which is significantly lower than the healthy control, 7.9 ± 0.9/mm (p < 0.0001). Despite the improvement in mean ERL count over time, it still remained below the expected normal level obtained in healthy controls (6.1–9.7/mm), even after five years of medical therapy (Fig. 2). The rate of improvement varied depending on the different disease courses, with the monocyclic short disease course showing more change than the other groups (4.8 ± 1.5/mm at baseline vs. 6.7 ± 1.5/mm at 12 months, p = 0.038 paired T-test).

**Fig. 3:**
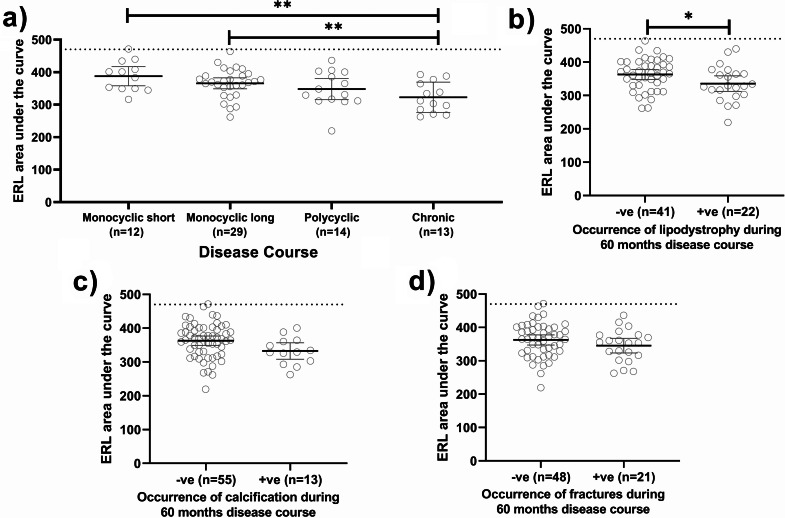
Disease courses and complications vs. ERL area under the curve (AUC). **(a)** There is a significant difference between the AUC for monocyclic short vs. chronic course as well as a significant difference between monocyclic long vs. chronic disease course, both p < 0.01. **(b)** Lipodystrophy of any type (generalized, partial, or localized) has lower ERL AUC, p = 0.04 than JDM without lipodystrophy. **(c)** There are no associations of ERL AUC with calcifications. **(d)** There are no associations of ERL AUC with fractures

To evaluate the accumulative effects of chronic ERL capillary loss, the ERL AUC was calculated for each patient (Fig. 1). Although there was a positive correlation between the initial ERL count and ERL AUC, the correlation was not strong (r^2^ = 0.18, p = 0.001). There was a significant difference between the ERL AUC for monocyclic short vs. chronic (389 ± 46.46 vs. 313 ± 46.69, p = 0.001) and monocyclic long vs. chronic (359 ± 44.53 vs. 313 ± 46.69, p = 0.013) (Fig. 3a). Next, the relationship between ERL AUC and indicators of disease damage (lipodystrophy and calcification) was evaluated. JDM patients with lipodystrophy exhibited a lower ERL AUC than those without lipodystrophy (335.7 ± 52.52 vs. 363.0 ± 47.13, p = 0.04) (Fig. 3b). However, the ERL AUC had no significant association with calcifications (Fig. 3c). Lastly, the relationship between ERL AUC and fractures was assessed, revealing no significant correlation (Fig. 3d).

**Fig. 4:**
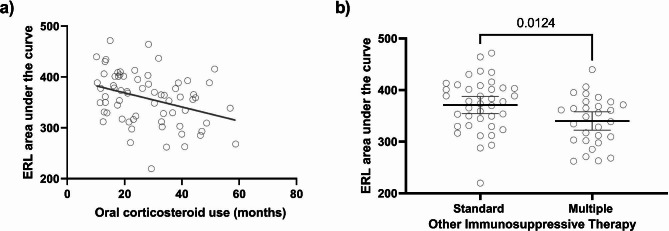
Medication use and ERL area under the curve (AUC). **a**) There was a negative correlation between the duration of oral steroid use in months and ERL AUC (r^2^ = 0.12, p = 0.004). **b**) JDM children who received standard immunosuppressive therapy (steroid, hydroxychloroquine, and methotrexate) had a higher ERL AUC than those who were given multiple immunosuppressive medications (339.9 ± 46.8 vs. 371.0 ± 51.0, p = 0.01)

Next, we evaluated the effect of medical treatment on ERL AUC. There was a negative correlation between the duration of oral steroid use and ERL AUC (r^2^ = 0.12, p = 0.004). Patients who required multiple immunosuppressive medications (additional immunosuppression more than steroid, hydroxychloroquine, and methotrexate) tended to have a lower ERL AUC (339.9 ± 46.8 vs. 371.0 ± 51.0, p = 0.01) (Fig. 4).

## Discussion

This study provides insight into the association between endothelial dysfunction demonstrated by decreased ERL count and disease progression in children with JDM. Consistent with previous studies, we observed a significantly lower mean ERL density in untreated JDM children compared to healthy controls [6, 7]. This finding supports the concept of endothelial involvement in JDM pathophysiology [3]. Furthermore, the reduction in ERL count often correlates with the severity of skin disease and/or muscle weakness [6, 17], suggesting its possible role as a potential physical exam indicator ofr disease activity. Few studies have evaluated the changes in capillary density and disease activity longitudinally over the disease course [17, 18]. We observed variations in the rate of improvement in ERL count in the different JDM disease courses. Monocyclic disease courses show improvement of the ERL capillary count at a faster rate than that of chronic disease. This finding suggests that the rate of improvement in ERL count may be a more critical factor than the initial ERL count in predicting disease course and outcome.

Despite medical therapy, the mean ERL count in JDM patients remained below normal levels even after five years of treatment, particularly in the polyphasic and chronic disease courses. This implies that the restoration of capillary density might be challenging to achieve and may require additional novel therapeutic strategies to target endothelial dysfunction effectively [19].

Additionally, we utilized the AUC method to evaluate the cumulative change in ERL capillary density over the study period. Our results demonstrated a correlation between ERL AUC and a more chronic disease course, as well as the presence of complications such as lipodystrophy. Of note, we found a weakly positive correlation between the initial ERL count and ERL AUC, which suggests that factors other than the initial capillary density, including MSAs, may contribute to the cumulative capillary loss. For example, it has been shown JDM with anti-P155/140 antibody tend to have lower ERL capillary count and are less likely to have a monophasic disease course [5]. However, our study was not powered enough to investigate the impact of the type or the duration of different MSAs on ERL AUC.

Circulating endothelial cells and markers of endothelial injury, such as vWF antigen, and thrombomodulin are elevated in JDM, providing further evidence of endothelial involvement in the disease pathophysiology [8, 19, 20]. B cell activation and expansion as well as the formation of anti-endothelial cell antibodies have been demonstrated in JDM [21–24], suggesting other potential mechanisms for endothelial cell injury. Soluble adhesion molecule markers, such as ICAM-1, ICAM-3, and VCAM1, and inflammatory cytokine and markers like neopterin have been used as possible biomarkers of vasculopathy in JDM [25–27]. These findings highlight the complex interplay between immune dysregulation and endothelial dysfunction in JDM, requiring further investigation to elucidate the underlying mechanisms. Furthermore, it is important to recognize the clinical implications of microvascular injury in JDM as it can affect the gastrointestinal system [28]. The reduced nailfold capillary density observed in JDM has been associated with impaired absorption of oral prednisone, potentially leading to suboptimal drug levels and ineffective treatment [9]. Therefore, administering medications by the intravenous or subcutaneous routes might be preferred in patients with persistently low ERL counts.

The study has several limitations. First, the sample size is relatively small, particularly when considering the potential heterogeneity within the JDM population. Second, the diagnosis of lipodystrophy was based on physician assessment, which can introduce some subjectivity. Lastly, the study was conducted at a single center, potentially limiting the generalizability, especially in geographic regions where P155/140 autoantibodies are not the predominant autoantibody.

## Conclusions

Persistently decreased ERL capillary density documented by low ERL AUC is associated with both a chronic disease course and lipodystrophy in JDM. Despite medical therapy, the mean ERL count remained below normal, even after five years, particularly in polycyclic and chronic cases. Therefore, restoring normal ERL capillary density might be challenging and require novel therapeutic strategies targeting endothelial dysfunction.

### Electronic supplementary material

Below is the link to the electronic supplementary material.

Supplementary Material 1: Table 1. Demographics of healthy controls (n = 77)

## Data Availability

The data that support the findings of this study are available from the corresponding author, [LMP], upon reasonable request.

## Abbreviations

JDM: Juvenile Dermatomyositis
ERL: End row loops
AUC: Area under the curve
vWF antigen: von Willebrand factor antigen
IV: Intravenous
DAS: Disease Activity Score
CMAS: Childhood Myositis Assessment Scale
MSA: Myositis-specific antibodies
