# Supplementary material for: Juvenile dermatomyositis: association between nail fold capillary end row loop– area under the curve– and disease damage indicators
Source: Pediatr Rheumatol Online J. 2023 Nov 13;21:137. doi: 10.1186/s12969-023-00919-3 (PMC10641947; doi:10.1186/s12969-023-00919-3)
Supplement: Supplementary file 1 — Supplementary Material 1: Table 1. Demographics of healthy controls (n = 77) [file 12969_2023_919_MOESM1_ESM.docx]

**Supplemental Table 1**: Demographics of healthy controls (n=77).

|  | Frequency (n) | Percentage |
| --- | --- | --- |
| Sample size | 77 |  |
| Sex |  |  |
| Female | 35 | 45.5% |
| Male | 42 | 54.5% |
| Race/Ethnicity |  |  |
| White | 40 | 51.9% |
| Hispanic | 19 | 24.7% |
| African American | 4 | 5.2% |
| Others | 10 | 13% |
| Unknown | 4 | 5.2% |
|  | *Mean ± SD* | *Median (range)* |
| Age (years) | 8.8 ± 4.3 | 8.7 (2.58-17.7) |
| ERL (#/mm) | 7.9 ± 0.9 | 7.9 (6.1-11.5) |
